# A Standardised Car Seat Transfer Test: Reliability and Concurrent Validity

**DOI:** 10.1155/oti/7306142

**Published:** 2025-07-14

**Authors:** Tarcisio F. de Campos, Mitchell Wolden, Marie K. March, Deshitha Hewawasam, Brandon Boumelhem, Jessica J. Spurr, James M. Khoury, Nick Vertzyas, Steven G. Faux, Gregory C. Gass, Sean F. Mungovan

**Affiliations:** ^1^JW & M Cunningham Orthopaedic Clinical Research Fellow, St Vincent's Private Hospital, Sydney, New South Wales, Australia; ^2^St Vincent's Private Allied Health Services, St Vincent's Private Hospital, Sydney, New South Wales, Australia; ^3^Physical Therapy Program, University of Jamestown, Fargo, North Dakota, USA; ^4^The Clinical Research Institute, Sydney, New South Wales, Australia; ^5^Faculty of Medicine and Health, University of Sydney, Camperdown, New South Wales, Australia; ^6^Department of Orthopaedic Surgery, St Vincent's Private Hospital, Sydney, New South Wales, Australia; ^7^School of Medicine, University of Notre Dame, Sydney, New South Wales, Australia; ^8^Department of Rehabilitation Medicine, St Vincent's Private Hospital Sydney, Sydney, Australia; ^9^St Vincent's Clinical School, University of New South Wales, Sydney, Australia; ^10^School of Health and Biomedical Sciences, Royal Melbourne Institute of Technology (RMIT) University, Melbourne, Victoria, Australia

**Keywords:** activities of daily living, automobiles, car transfer, reliability, validity

## Abstract

**Introduction:** Transferring effectively into and out of a vehicle seat is required for functional mobility and social participation. Reliable and valid vehicle and simulator car seat transfer tests are limited. The aim of this study was to assess the reliability and concurrent validity of a car seat transfer test for application with a vehicle and a simulator car seat.

**Methods:** A cross-sectional study was conducted and reported according to the Guidelines for Reporting Reliability and Agreement Studies (GRRAS), the Strengthening the Reporting of Observational Studies in Epidemiology (STROBE) statements and the National Statement on Ethical Conduct in Human Research 2007. Consecutive healthy adults who expressed interest and met the inclusion criteria were recruited for our study. A standardised vehicle and simulator car seat transfer test was undertaken on two visits, 7 days apart. WOMAC, hip and knee active range of motion assessment, self-selected gait speed measurement and the five-repetition sit-to-stand test were undertaken on the first visit.

**Results:** Complete data collection was performed with 42 healthy adults. There was good interrater (ICC = 0.99; 95% CI 0.99, 1.00), intrarater (ICC = 0.79; 95% CI 0.62, 0.89 to ICC = 0.91; 95% CI 0.76, 0.96) and test–retest reliability (ICC = 0.87; 95% CI 0.77, 0.93 to ICC = 0.94; 95% CI 0.89, 0.96) for car seat transfer test times for getting into and out of the vehicle and the simulator car seat. There were strong and significant correlations (*r* = 0.84–0.89) between the vehicle and simulator car seat test times; these times correlated significantly with measures of physical function including self-selected gait speed and five-repetition sit-to-stand test results (*p* < 0.001).

**Conclusion:** A standardised car seat transfer test is reliable and valid for testing car seat transfer ability in a vehicle and a simulator car seat.

**Summary**

• This study supports the reliability and concurrent validity of a simulator car seat transfer test.
• We recommend the mean of three car seat transfer tests as the test result.
• Separately measuring times for getting into and out of a car seat may help assess car seat transfer ability.

## 1. Introduction

For older individuals, successfully transferring into and out of a vehicle is essential for maintaining autonomy, accessing the community, engaging in social interactions and preserving quality of life [1–3]. Car seat transfers require an integrated combination of physical and cognitive abilities, including lower limb strength, sufficient hip and knee range of motion, balance and executive function, all of which decline with healthy ageing [4–7]. As a result, performing the coordinated movements to safely and successfully get into and out of a car seat can become a significant barrier to mobility, independence and social participation later in life [8].

Occupational therapists routinely assess car seat transfer ability to inform rehabilitation goal and discharge planning [9]. The car seat transfers are often assessed using a dedicated vehicle. However, not all occupational therapy clinical settings have access to a dedicated vehicle due to space, cost or safety concerns [10, 11]. An alternative for assessing car seat transfer ability in older individuals is the use of a simulator car seat; however, the clinical utility requires that the simulator-based assessments are both reliable and comparable to those obtained with a real vehicle.

Despite the routine clinical assessment of car seat transfer ability in older individuals, no studies to date have evaluated the reliability and concurrent validity of a standardised car seat transfer test applied to both a vehicle and simulator car seat. The absence of established psychometric properties that give insight into the quality and effectiveness of car seat transfer tests with a vehicle and simulator is a critical gap in the literature. Without established reliability and concurrent validity, occupational therapists should have limited confidence that simulator-based assessments accurately reflect real-world performance, potentially limiting their use in clinical decision-making.

Our aim was to assess the reliability and concurrent validity of a standardised test to assess car seat transfer ability in healthy adults, using a vehicle and a simulator car seat. Establishing the psychometric properties of a standardised car seat transfer test is an important next step for improving functional mobility assessments and supporting evidence-based practise in occupational therapy.

## 2. Methods

### 2.1. Study Design

A cross-sectional study was conducted and reported according to the Guidelines for Reporting Reliability and Agreement Studies (GRRAS; supplemental checklist 1), the Strengthening the Reporting of Observational Studies in Epidemiology (STROBE; supplemental checklist 2) statements and the National Statement on Ethical Conduct in Human Research 2007 [12, 13]. Ethical approval was obtained from St Vincent's Hospital Human Research Ethics Committee (Ref. Number: 2021/ETH11381). All eligible healthy adult participants were informed about the objectives and procedures of the study and provided their written consent. The outline of the study design is shown in Figure S1.

### 2.2. Study Setting and Participants

The study population was recruited from the local community through flyers and advertisements posted in the Allied Health Department, St Vincent's Private Hospital, Sydney and the Department of Orthopaedics, St Vincent's Clinic, Sydney, Australia. From 14 June to 28 September 2022. Consecutive healthy adults who expressed interest in participating in our study were contacted to discuss the details of the study and assessed for eligibility. The inclusion criteria were as follows: (i) age 50 years or older, (ii) able to get in and out of a car seat independently, and (iii) proficient in reading and understanding English. The exclusion criteria were as follows: (i) a history of diagnosed hip and/or knee osteoarthritis and/or hip or knee arthroplasty; (ii) a known musculoskeletal, neurological and/or cardiovascular comorbid condition that could impact the assessment procedures; (iii) a psychiatric or psychological disorder; and (iv) a body mass index (BMI) over 35 kg/m^2^. Data collection took place in locations used for routine clinical care at St Vincent's Private Hospital, Sydney, Australia.

### 2.3. Study Procedures

To assess the reliability and concurrent validity of a car seat transfer test applied to a vehicle and simulator car seat, participants completed testing at an initial visit (Visit 1) and repeated on a separate visit 7 days later (Visit 2).

#### 2.3.1. Visit 1

At Visit 1, participant characteristics including sociodemographic, anthropometric (height, weight, BMI and leg length), active hip and knee joint range of motion and the Western Ontario and McMaster University (WOMAC) Osteoarthritis Index [14] were obtained. Participants then completed three vehicle and three simulator car seat transfer tests. Randomisation of the vehicle and simulator car seat transfer testing was performed using an online random sampling website (https://www.randomizer.org). Following the vehicle and simulator car seat transfer tests, participants completed measures of physical function including a standardised self-selected gait assessment using the GAITRite system and a five-repetition sit-to-stand (5STS) test [15].

#### 2.3.2. Visit 2

At Visit 2, participants completed the three vehicle and three simulator car seat transfer tests. Randomisation of the vehicle and simulator car seat transfer testing was also performed using the same online random sampling website.

### 2.4. Car Seat Transfer Testing Procedures

#### 2.4.1. Vehicle and Simulator

**Figure 1:**
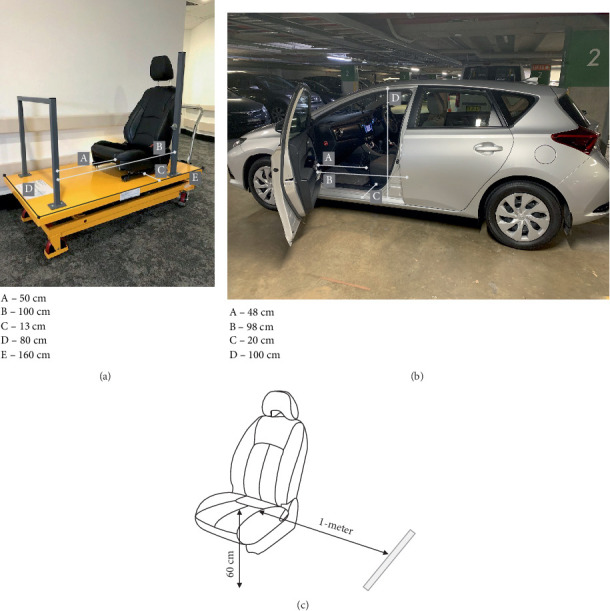
(a) Simulator car seat and vehicle dimensions. (b) Vehicle dimensions. (c) The experimental set-up for the vehicle and simulator car seat transfer test protocol.

Our study used a Toyota Corolla Sedan 2017 vehicle, situated in the St Vincent's Private Hospital car park, and a simulator car seat situated in the Allied Health Department at St Vincent's Private Hospital, Sydney and the St Vincent's Clinic, Sydney (Figure 1a,b).

### 2.5. Car Seat Transfer Testing Protocol

Participants were instructed to wear comfortable clothing and closed shoes that corresponded to what they would wear when getting in and out of a vehicle. All research team members were trained to carry out the test protocol and all other assessments in this study. One researcher provided instructions to participants on how to perform the car seat transfer testing procedures and gave a demonstration that included getting into and out of the vehicle and simulator car seat. Participants then practised getting into and out of the vehicle and simulator car seat twice prior to formal testing. Three randomly assigned vehicle and simulator car seat transfer tests were then completed. Two independent blinded researchers recorded the time taken by each participant to get into and out of the vehicle and simulator car seat using a hand-held stop watch (HART Sport).

Participants began the car seat transfer test protocol in a standardised starting position facing the side of the vehicle or the simulator car seat with their feet positioned on a marked line 1 m from the side of the vehicle or simulator car seat (Figure 1c). Before commencing the vehicle and simulator test, participants received the following instructions: *“This is a test in which we would like to assess your ability to safely get into and out of a car seat. The test has two components. First, we will ask you to get into the car seat, and then we will ask you to get out of the car seat*.”

#### 2.5.1. Getting Into the Car Seat

Participants were first instructed to get into the vehicle or simulator car seat by a member of the research team using the following instructions: *“We would now like to assess your ability to safely get into the car seat. When I say ‘Ready, go', please get into the car seat, at a comfortable pace, and place both feet on the floor of the vehicle or the simulator.”* The member of the research team conducting the car seat transfer test then gave the command *“Ready, go”.* Two researchers (Rater 1 and Rater 2) timed the test using a handheld stopwatch (HART Sport). The two researchers started the stopwatches when the participant's foot began to move to undertake the test and stopped when the participant's feet were positioned on the floor of the vehicle or on the platform of the simulator. The time recorded by the two researchers was then entered into the participant's study handbook.

#### 2.5.2. Getting Out of the Car Seat

Participants were instructed to get out of the vehicle and simulator car seat by a member of the research team using the following instructions: “*We would now like to assess your ability to safely get out of the car seat. When I say ‘Ready, go, please get out of the car seat at a comfortable pace, and place both feet on the marked starting line.*” The member of the research team conducting the test then gave the command “Ready, go”. The two researchers timing the test started their stopwatches when the participant's foot began to move off the floor of the vehicle or off the platform of the simulator and stopped timing when the participant was in a fully upright standing position with both feet positioned on the marked line 1 m from the side of the car seat. The times recorded by the two researchers were entered into the participant's study handbook.

### 2.6. WOMAC

To assess lower limb pain, stiffness and physical function, participants completed the validated, self-administered WOMAC questionnaire. The WOMAC questionnaire is comprised of 24 items divided into three domains: pain (5 items; range 0–20), stiffness (2 items; range 0–8) and physical function (17 items; range 0–68). Each of the 24 items was rated by participants on a 5-point Likert scale from 0 to 4, where 0 corresponds to ‘*none*', 1 to ‘*mild*', 2 to ‘*moderate*', 3 to ‘*severe*' and 4 to ‘*extreme*'. Higher WOMAC questionnaire scores are indicative of greater pain and stiffness and lower levels of physical function. Lower WOMAC questionnaire scores suggest the absence of significant joint pain, stiffness and functional limitations related to osteoarthritis and/or other musculoskeletal conditions.

### 2.7. Gait Assessment

The gait assessment protocol involved walking a distance of 10 m at a self-selected gait speed that included walking over the 4.6-m instrumented GAITRite system walkway mat. Each participant completed two practise passes over the GAITRite system walkway mat then three successive formal gait-testing passes. To ensure that acceleration and deceleration occurred beyond the borders of the GAITRite walkway mat, each trial started 2 m in front of the walkway and finished 2 m beyond the end of the walkway. Participants were instructed to start walking from a marked starting line on the command “Ready, go” and to continue walking until they reached the marked finish line positioned 2 m beyond the end of the walkway. Data from the three successive passes over the GAITRite system walkway mat were averaged for each participant to define the individual gait assessment parameters including left and right step length (centimetre) and self-selected gait speed (metre per second) [16].

### 2.8. 5STS Test

Lower limb functional capacity was measured using two 5STS tests. Participants were asked to stand up and sit down from a 48-cm chair five times, without using their arms and as fast as possible. The fastest time of the two 5STS tests was recorded for subsequent analysis [15].

### 2.9. Outcome Measures

The primary outcome measure was the time (seconds) to get into and out of the vehicle and simulator car seat. Secondary outcomes included self-selected gait speed (meter per second), 5STS test (seconds) and the pain (0–20), stiffness (0–8) and physical function (0–68) domains of the WOMAC.

### 2.10. Statistical Methods

#### 2.10.1. Sample Size Estimation and Justification

To estimate the required sample size, we applied the methods described by Walter et al. [17] assuming two replications, 80% power and a significance level of 5%. In the absence of prior pilot data or published ICC values for similar assessments, we selected an expected ICC of 0.60 as a conservative estimate to reflect moderate reliability. An alternative hypothesis ICC of 0.80 was used to represent a meaningful improvement in reliability [17]. The analysis determined that 39 participants were required. To account for a possible dropout rate (10%), an additional four participants were recruited, resulting in a projected sample size of 43 participants.

### 2.11. Data Analysis

Data analysis was performed using the STATA/MP 17.0 (StataCorp, Texas, United States) statistical software package (StataCorp). The statistical significance level was set as *p* < 0.05 for all results. Demographic data and continuous variables were analysed by calculating the means, standard deviation (SD) and 95% confidence interval (95% CI). The assumption of normality was evaluated using the Shapiro–Wilk test. The differences in time (seconds) between getting in and out of the vehicle and the simulator car seat were assessed using independent sample *t*-tests.

#### 2.11.1. Interrater, Intrarater and Test–Retest Reliability

Assessment of the interrater, intrarater and test–retest reliability of the vehicle and simulator car seat transfer tests for Visit 1 and Visit 2 was performed using an ICC with a 95% CI [18]. The ICC estimates and the 95% CIs were calculated for all reliability assessments based on an absolute-agreement, random-effects model [19]. The 95% CIs of ICC values were interpreted to evaluate the level of reliability as follows: poor (<0.5), moderate (0.5–0.75), good (0.76–0.9) and excellent (>0.9) [20].

#### 2.11.2. Standard Error of Measurement (SEM) and Minimal Detectable Change (MDC)

Absolute reliability was determined by calculating SEM and MDC at 90% CI (MDC_90_) for each measurement. The SEM reflects the measurement error for a given technique and was calculated using the following equation: SEM = SD√(l–ICC) [21]. To determine the magnitude of change that exceeded the threshold of measurement error at a 90% confidence level, the MDC_90_ was calculated using the formula (MDC_90_) = 1.645∗SEM∗square root of 2 [21].

#### 2.11.3. Concurrent Validity

Pearson–product moment correlation coefficients (*r*) were used to estimate the concurrent validity of the vehicle and simulator transfer test times. Interpretation of Pearson's *r* was: weak (<0.4), moderate (0.4–0.7), and strong (>0.7) [22]. Bland–Altman plots were constructed to assess bias and the 95% CI for the limits of agreement (LOAs) between measurements of the vehicle and the simulator car seat transfer test times during Visit 1 and Visit 2 [23].

## 3. Results

### 3.1. Flow of Participants Through the Study

A total of 52 adults were screened for eligibility; six declined to participate, and four were deemed ineligible (three before Visit 1 and one before the completion of Visit 2). Data from 42 participants across Visit 1 and Visit 2 were analysed (Figure S1).

### 3.2. Characteristics of the Study Participants and Assessors

Then, 42 participants with a mean age of 65 years (SD 8) were included in the study; 24 (57%) were female and 18 (43%) were male. The mean height, weight and BMI were 168 cm (SD 10), 75.8 kg (SD 16.8) and 26.6 kg/m^2^ (SD 4.3), respectively.  Table 1 presents the characteristics of the participants included in the study. Eight assessors (four occupational therapists and four physiotherapists) were involved in the data collection procedures.

### 3.3. Car Seat Transfer Assessment Results

The Visit 1 and Visit 2 vehicle and simulator car seat transfer test results are listed in Table 2. Shapiro–Wilk tests for normality were conducted on all continuous variables yielding nonsignificant results. Data analysis proceeded under the assumption of a normal distribution for each variable.

### 3.4. Reliability

#### 3.4.1. Interrater Reliability

There was excellent (ICC = 0.99; 95% CI 0.99, 1.00) interrater reliability for test times getting into and out of the car seat with the vehicle as well as with the simulator (Table 3).

#### 3.4.2. Intrarater Reliability

For the analysis of intrarater reliability, we included data from 35 participants who had the same assessor for Visit 1 and Visit 2. Intrarater reliability was moderate to excellent for times getting into (ICC_vehicle_ = 0.91; 95% CI 0.76, 0.96 and ICC_simulator_ = 0.79; 95% CI 0.62, 0.89) and out of (ICC_vehicle_ = 0.87; 95% CI 0.64, 0.95 and ICC_simulator_ = 0.81; 95% CI 0.65, 0.90) both the vehicle and the simulator car seat (Table 4).

#### 3.4.3. Test–Retest Reliability

There was good to excellent test–retest reliability for times getting into (ICC_vehicle_ = 0.94; 95% CI 0.89, 0.96 and ICC_simulator_ = 0.87; 95% CI 0.77, 0.93) and out of (ICC_vehicle_ = 0.91; 95% CI 0.83, 0.95 and ICC_simulator_ = 0.88; 95% CI 0.79, 0.94) the car seat using both the vehicle and the simulator (Table 5).

### 3.5. Concurrent Validity

There were strong and significant correlations (*r* = 0.84–0.89) between the vehicle and simulator car seat test results at Visit 1 and Visit 2 for getting into the car seat and getting out of the car seat (Table 6). Normal distribution of differences was assumed, as most of the differences fell within the 95% LOA [23]. The simulator car seat transfer test times were significantly shorter than the vehicle transfer test times (seconds) at Visit 1 and Visit 2 for getting into the car seat (Visit 1: 0.15, LOA 95%, −0.58 to 0.88; Visit 2: 0.11, LOA 95%, −0.55 to 0.76, *p* < 0.05) and getting out of the car seat (Visit 1: 0.42, LOA 95% −0.46 to 1.30; Visit 2: 0.26, LOA 95% −0.49 to 1.00) (Figures S2, S3, S4, and S5).

### 3.6. Correlation With Physical Function Measures

#### 3.6.1. Self-Selected Gait Speed

There were significant and moderate negative correlations (*r* = −0.43–−0.54) between self-selected gait speed and the vehicle and simulator car seat transfer test times (*p* < 0.001) (Table 7).

#### 3.6.2. WOMAC

There were no significant correlations between the three domains of the WOMAC (pain, stiffness and physical function) and the vehicle and simulator car seat transfer test times (Table 7).

#### 3.6.3. 5STS Test

There were significant and moderate-to-strong positive correlations (*r* = 0.51–0.65) between the 5STS test and the vehicle and simulator car seat transfer test times (*p* < 0.001) (Table 7).

## 4. Discussion

The present study investigated the reliability and concurrent validity of a standardised car seat transfer test protocol, applied to a vehicle and a simulator car seat, in a group of healthy older adults. Our study found that a standardised car seat transfer test, designed to assess getting in and getting out of a car seat, is a reliable and valid method for evaluating car transfer ability using a vehicle or a simulator car seat. We report moderate-to-strong correlations of vehicle and simulator car seat transfer test times with measures of physical function, including the 5STS test time and self-selected gait speed.

To the best of our knowledge, our study is the first to establish both the reliability and concurrent validity of a standardised vehicle and simulator car seat transfer test protocol in healthy adults aged 50 years or older. Our study comprised healthy older adults without any reported hip or knee pathology; the lack of hip or knee pathology was supported by low WOMAC scores in all three domains (pain, stiffness and physical function) with normal hip and knee range of motion and functional testing outcomes including self-selected gait speed and 5STS test times [6, 24]. Although the car seat transfer test times were on average 0.13 s faster when getting into and 0.34 s faster when getting out of the simulator car seat compared to the vehicle, we consider these differences to be clinically insignificant.

Our reliability findings are consistent with those of others where participants with knee osteoarthritis completed a combined task of getting into and out of the simulator car seat as fast as possible, [25]. However, our novel testing protocol highlights an important distinction: The demands associated with getting into and out of a vehicle or simulator car seat include biomechanical and neuromuscular considerations associated with the descent and ascent of an individual's centre of mass [26]. Getting into a car seat involves eccentric lower limb muscular activity to control the descent of an individual's centre of mass from an upright position onto the car seat. Conversely, getting out of the car seat requires concentric lower limb muscle activity to elevate the centre of mass from the seat to an upright posture. From a neuromuscular perspective, getting into and out of a car seat requires controlled balance, sequenced coordination and proprioceptive feedback. The specific neuromuscular demands of getting into and out of a car seat vary depending on the direction of motion and the type of muscle activation. We selected to develop a self-paced testing protocol rather than a protocol requiring participants to get into and out of the vehicle or simulator car seat as quickly as possible. Self-paced testing provides a more realistic assessment of usual daily function and prioritises safety with the testing procedures, whilst maintaining reliability and validity [27, 28].

Our study incorporated a simulator car seat that replicated the characteristics of the vehicle used. The simulator car seat was affixed to a height adjustable platform, allowing for the simulator car seat to be positioned at the same height as the vehicle car seat. Although simulation has been widely used by occupational therapists for the assessment of functional tasks, including driving ability assessments [29, 30], the implementation of simulation for car seat transfer testing is underreported. Simulation for car seat transfer testing confers advantages, including (i) streamlined access to assessment within clinical settings, (ii) obviating the necessity for scheduling vehicle access and (iii) mitigating associated risks, such as those inherent in accessing vehicles located in car parks.

To the best of our knowledge, this is the first study to report the correlations of lower limb functional capacity assessment using the 5STS test and self-selected gait speed with car transfer ability amongst healthy adults. Future investigations will extrapolate our findings to specific patient cohorts for whom successful car transfers are a priority.

### 4.1. Strengths and Limitations

The strengths of our study include (i) a homogeneous sample of healthy adult volunteers, (ii) a standardised testing environment for assessment of car transfer ability with the vehicle and the simulator car seat across Visit 1 and Visit 2, (iii) adherence to a standardised test protocol that included the assessment of getting into and out of the vehicle and simulator car seat and (iv) occupational therapists and physiotherapists conducting the testing who had received protocol training. The limitations of our study include (i) the omission of opening and closing a car door and (ii) putting on and taking off a seat belt. Consequently, our results may not represent the full complexity of car seat transfer tasks. (iii) A third limitation for consideration is the uncertainty surrounding the power analysis used to determine the sample size. In the absence of prior data or pilot testing, we selected an expected ICC of 0.60 as a conservative estimate to guide our sample size calculation. Nevertheless, these limitations underscore potential avenues for future research to refine and expand our current understanding of car seat transfer assessments.

### 4.2. Implications for Practise

The findings from our study have clinical implications for practise including the following:

i. A vehicle and simulator car seat transfer test can be reliably used in clinical settings to assess healthy adults' ability to get into and out of a car seat.
ii. The test protocol should include formal familiarisation with the testing procedures and use the average of three trials as the test result.
iii. The use of a simulator car seat transfer that is height adjustable can increase access to valid and safe assessments of car transfer ability.

### 4.3. Future Directions

Whilst we determined that a standardised protocol to assess car seat transfer ability using a vehicle or a simulator car seat is valid and reliable in healthy adults, future research is required to investigate the vehicle and simulator car seat transfer test in specific patient populations who have limitations in completing car seat transfers.

## 5. Conclusions

A standardised car seat transfer test protocol that includes getting into and out of a car seat is reliable and valid for evaluating car transfer ability in healthy adults using a vehicle or a simulator car seat.

## Figures and Tables

**Table 1:** Characteristics and measures of physical function of the participants in the study.

**Characteristic**	**Participants (** **n** = 42**)**
Age, years, mean (SD)	65 (8)
Women, *n* (%), men, *n* (%)	24 (57), 18 (43)
Height (cm), mean (SD)	168 (10)
Weight (kg), mean (SD)	75.8 (16.8)
BMI (kg/m^2^), mean (SD)	26.6 (4.3)
Lower limb length (cm), mean (SD)	
Left	89 (5)
Right	89 (5)
Active joint range of motion (degrees), mean (SD)	
Standing hip flexion	
Left	103 (11)
Right	103 (10)
Seated knee flexion	
Left	123 (6)
Right	122 (7)
Supine knee flexion	
Left	135 (7)
Right	134 (7)
Physical function measures	
Gait parameters, mean (SD)	
Self-selected gait speed (m/s)	1.47 (0.19)
Step length (cm)	
Left	74.5 (6.7)
Right	74.4 (7.2)
Five-repetition sit-to-stand test (s), mean (SD)	9.8 (2.7)
WOMAC^†^, mean (SD)	
Pain	0.5 (1.2)
Stiffness	0.5 (0.9)
Difficulty performing daily activities	1.6 (3.7)

Abbreviations: %, percentage; BMI, body mass index; cm, centimetres; m, metres; min, minutes; *n*, number of participants included in the analyses; s, seconds; SD, standard deviation; WOMAC, Western Ontario and McMaster University Osteoarthritis Index.

^†^WOMAC, Western Ontario and McMaster University (WOMAC) Osteoarthritis Index: The pain domain score range from 0 (lower levels of symptoms) to 20 (extreme levels of symptoms), the stiffness domain score from 0 (lower levels of symptoms) to 8 (extreme levels of symptoms), and the difficulty performing daily activities domain score 0 (lower levels of disability) to 68 (extreme levels of disability).

**Table 2:** Reliability and concurrent validity of vehicle and simulator car seat transfer test. Mean (SD) (s) for the vehicle and simulator car seat transfer test times in healthy adults (*n* = 42).

**Variable**	**Time (s)**			
	**Visit 1**		**Visit 2**	
	**Assessor 1**	**Assessor 2**	**Assessor 1**	**Assessor 2**
Vehicle				
Getting into the car seat	3.42 (0.75)	3.40 (0.73)	3.22 (0.73)	3.24 (0.71)
Getting out of the car seat	3.38 (0.83)	3.35 (0.83)	3.10 (0.70)	3.14 (0.70)
Simulator				
Getting into the car seat	3.28 (0.59)	3.25 (0.58)	3.12 (0.59)	3.12 (0.61)
Getting out of the car seat	2.95 (0.55)	2.94 (0.56)	2.87 (0.59)	2.86 (0.58)

*Note*: Mean of three valid car seat transfer tests.

Abbreviations: CI, confidence interval; ICC, intraclass correlation coefficient; MDC_90_, minimal detectable change at 90% CI; *n*, refers to the total number of participants included in the analyses; s, seconds; SD, standard deviation; SEM, standard error of measurement.

⁣^∗^* p* value less than 0.05 for comparison of the vehicle and the simulator car seat transfer test times.

**Table 3:** Reliability and concurrent validity of vehicle and simulator car seat transfer test. Interrater reliability: ICC (95% CI) amongst raters for the car seat transfer test as applied using a vehicle and a simulator car seat in healthy adults (*n* = 42).

**Variable**	**Visit 1**			**Visit 2**		
	**ICC (95% CI)**	**SEM**	**MDC**_**90**_	**ICC (95% CI)**	**SEM**	**MDC**_**90**_
Vehicle						
Getting into the car seat	0.99 (0.99–1.00)	0.07	0.17	0.99 (0.99–1.00)	0.07	0.17
Getting out of the car seat	0.99 (0.99–1.00)	0.08	0.19	0.99 (0.99–1.00)	0.07	0.16
Simulator						
Getting into the car seat	0.99 (0.99–1.00)	0.06	0.14	0.99 (0.99–1.00)	0.06	0.14
Getting out of the car seat	0.99 (0.99–1.00)	0.06	0.13	0.99 (0.99–1.00)	0.06	0.1

*Note*: Mean of three valid car seat transfer tests.

Abbreviations: CI, confidence interval; ICC, intraclass correlation coefficient; MDC_90_, minimal detectable change at 90% CI; *n*, refers to the total number of participants included in the analyses; s, seconds; SD, standard deviation; SEM, Standard error of measurement.

⁣^∗^* p* value less than 0.05 for comparison of the vehicle and the simulator car seat transfer test times.

**Table 4:** Reliability and concurrent validity of vehicle and simulator car seat transfer test. Intrarater reliability: ICC (95% CI) of the assessors for the car seat transfer test applied using a vehicle and simulator car seat in healthy adults (*n* = 35)^†^.

**Variable**	**ICC (95% CI)**	**SEM**	**MDC**_**90**_
Vehicle			
Getting into the car seat	0.91 (0.76–0.96)	0.22	0.52
Getting out of the car seat	0.87 (0.64–0.95)	0.27	0.62
Simulator			
Getting into the car seat	0.79 (0.62–0.89)	0.29	0.66
Getting out of the car seat	0.81 (0.65–0.90)	0.26	0.60

*Note*: Mean of three valid car seat transfer tests.

Abbreviations: CI, confidence interval; ICC, intraclass correlation coefficient; MDC_90_, minimal detectable change at 90% CI; *n*, the total number of participants included in the analyses; s, seconds; SD, standard deviation; SEM, standard error of measurement.

^†^We included data from 35 participants who had the same assessor for Visit 1 and Visit 2 for the analysis of intrarater reliability.

⁣^∗^* p* value less than 0.05 for comparison of the vehicle and the simulator car seat transfer test times.

**Table 5:** Reliability and concurrent validity of vehicle and simulator car seat transfer test. Test–retest reliability: ICC (95% CI) for the car seat transfer test as applied using a vehicle and simulator car seat in healthy adults (*n* = 42).

**Variable**	**ICC (95% CI)**	**SEM**	**MDC**_**90**_
Vehicle			
Getting into the car seat	0.94 (0.89–0.96)	0.16	0.38
Getting out of the car seat	0.91 (0.83–0.95)	0.20	0.47
Simulator			
Getting into the car seat	0.87 (0.77–0.93)	0.21	0.50
Getting out of the car seat	0.88 (0.79–0.94)	0.20	0.46

*Note*: Mean of three valid car seat transfer tests.

Abbreviations: CI, confidence interval; ICC, intraclass correlation coefficient; MDC_90_, minimal detectable change at 90% CI; *n*, the total number of participants included in the analyses; s, seconds; SD, standard deviation; SEM, standard error of measurement.

⁣^∗^* p* value less than 0.05 for comparison of the vehicle and the simulator car seat transfer test times.

**Table 6:** Reliability and concurrent validity of vehicle and simulator car seat transfer test. Concurrent validity between the vehicle and simulator car seat transfer test times (s) (*n* = 42).

**Variable**	**Visit 1**		**Visit 2**	
	**Mean (SD)**	**Pearson's ** **r**	**Mean (SD)**	**Pearson's ** **r**
Getting Into the car seat				
Vehicle	3.41 (0.74)	0.87	3.23 (0.72)	0.89
Simulator	3.26 (0.59)⁣^∗^		3.12 (0.60)⁣^∗^	
Getting out of the car seat				
Vehicle	3.37 (0.83)	0.86	3.12 (0.70)	0.84
Simulator	2.95 (0.55)⁣^∗^		2.86 (0.59)⁣^∗^	

*Note*: Mean of three valid car seat transfer tests.

Abbreviations: CI, confidence interval; ICC, intraclass correlation coefficient; MDC_90_, minimal detectable change at 90% CI; *n*, total number of participants included in the analyses; s, seconds; SD, standard deviation; SEM, standard error of measurement.

⁣^∗^* p* value less than 0.05 for comparison of the vehicle and the simulator car seat transfer test times.

**Table 7:** Pearson correlations (*r*) between vehicle and simulator car seat transfer test times and physical function measures (*n* = 42).

	**5STS test**	**Self-selected gait speed**	**Pain**	**WOMAC stiffness**	**Function**
Getting in the car					
Vehicle	0.54⁣^∗^	−0.43⁣^∗^	0.30	0.13	0.22
Simulator	0.51⁣^∗^	−0.44⁣^∗^	0.24	0.08	0.20
Getting out of the car					
Vehicle	0.62⁣^∗^	−0.54⁣^∗^	0.24	0.02	0.15
Simulator	0.65⁣^∗^	−0.46⁣^∗^	0.19	−0.06	0.08

Abbreviations: 5STS test, five-repetition sit-to-stand test; WOMAC, Western Ontario and McMaster Universities Osteoarthritis Index.

⁣^∗^*p* value < 0.001.

## Data Availability

The data that support the findings of this study are available on request from the corresponding author. The data are not publicly available due to privacy or ethical restrictions.

## Supporting Information

Supporting Information Additional supporting information can be found online in the Supporting Information section. Figure S1. Study design flow diagram. Displays the number of participants assessed for eligibility through the number of participants that completed the study. Figure S2. Bland–Altman plot of the vehicle and the simulator getting into the car seat transfer test times, reported in seconds, for Visit 1. The mean of the vehicle and the simulator getting into the car seat transfer test times was plotted against the difference of the vehicle and the simulator getting into the car seat transfer test times for each participant. The centre line displays the mean difference for the participants between the vehicle and the simulator car seat transfer tests. The 95% confidence intervals are shaded in grey. Figure S3. Bland–Altman plot of the vehicle and the simulator getting out of the car seat transfer test times, reported in seconds, for Visit 1. The mean of the vehicle and the simulator getting into the car seat transfer test times was plotted against the difference of the vehicle and the simulator getting into the car seat transfer test times for each participant. The centre line displays the mean difference for the participants between the vehicle and the simulator car seat transfer tests. The 95% confidence intervals are shaded in grey. Figure S4. Bland–Altman plot of the vehicle and the simulator getting into the car seat transfer test times, reported in seconds, for Visit 2. The mean of the vehicle and the simulator getting into the car seat transfer test times was plotted against the difference of the vehicle and the simulator getting into the car seat transfer test times for each participant. The centre line displays the mean difference for the participants between the vehicle and the simulator car seat transfer tests. The 95% confidence intervals are shaded in grey. Figure S5. Bland–Altman plot of the vehicle and the simulator getting out of the car seat transfer test times, reported in seconds, for Visit 2. The mean of the vehicle and the simulator getting into the car seat transfer test times was plotted against the difference of the vehicle and the simulator getting into the car seat transfer test times for each participant. The centre line displays the mean difference for the participants between the vehicle and the simulator car seat transfer tests. The 95% confidence intervals are shaded in grey.
