# Supplementary material for: A Standardised Car Seat Transfer Test: Reliability and Concurrent Validity
Source: Occup Ther Int. 2025 Jul 14;2025:7306142. doi: 10.1155/oti/7306142 (PMC12279433; doi:10.1155/oti/7306142)
Supplement: Supporting Information — Additional supporting information can be found online in the Supporting Information section. Figure S1. Study design flow diagram. Displays the number of participants assessed for eligibility through the number of participants that completed the study. Figure S2. Bland–Altman plot of the vehicle and the simulator getting into the car seat transfer test times, reported in seconds, for Visit 1. The mean of the vehicle and the simulator getting into the car seat transfer test times was plotted against the difference of the vehicle and the simulator getting into the car seat transfer test times for each participant. The centre line displays the mean difference for the participants between the vehicle and the simulator car seat transfer tests. The 95% confidence intervals are shaded in grey. Figure S3. Bland–Altman plot of the vehicle and the simulator getting out of the car seat transfer test times, reported in seconds, for Visit 1. The mean of the vehicle and the simulator getting into the car seat transfer test times was plotted against the difference of the vehicle and the simulator getting into the car seat transfer test times for each participant. The centre line displays the mean difference for the participants between the vehicle and the simulator car seat transfer tests. The 95% confidence intervals are shaded in grey. Figure S4. Bland–Altman plot of the vehicle and the simulator getting into the car seat transfer test times, reported in seconds, for Visit 2. The mean of the vehicle and the simulator getting into the car seat transfer test times was plotted against the difference of the vehicle and the simulator getting into the car seat transfer test times for each participant. The centre line displays the mean difference for the participants between the vehicle and the simulator car seat transfer tests. The 95% confidence intervals are shaded in grey. Figure S5. Bland–Altman plot of the vehicle and the simulator getting out of the car seat transfer test times, [file 7306142.f1.zip › Supplementary Figures 1 to 5.docx]

**Supplementary Figure 1.** Study design flow diagram

Adults assessed for eligibility (n=52)

Excluded (n=9)

- Not meeting inclusion criteria (n=3)
- Declined to participate (n=6)

## Enrolment

Included healthy adults (total n=43)

## Visit 1

Healthy adults (n=43)

- Screening form
- Consent form
- Demographics
- Outcome Assessments: WOMAC, gait assessment, vehicle car seat transfer tests, simulator car seat transfer tests^†^.

Healthy adults (n=43)

- Outcome Assessments: Vehicle car seat transfer tests, simulator car seat transfer tests.*

## Visit 2

Discontinued study visit 2 (n=1)

- Adult with a BMI >40 Kg/m^2^

Healthy adults (n=42)

- Inter-rater, Intra-rater, Test-retest Reliability
- SEM and MDC_90_
- Concurrent Validity

## Data analysis

n, number of participants; WOMAC, Western Ontario and McMaster University Osteoarthritis Index (WOMAC); BMI, Body Mass Index; SEM, standard error of measurement; MDC_90_, minimal detectable change at 90% confidence intervals.

^†^Randomly assigned vehicle and car seat simulator tests

| **Supplement Figure 2.** Bland-Altman plots of the vehicle and the simulator getting into the car seat transfer test times, reported in seconds, for the visit 1. The mean of the vehicle and the simulator getting into the car seat transfer test times was plotted against the difference of the vehicle and the simulator getting into the car seat transfer test times for each participant.  The centre line displays the mean difference for the participants between the vehicle and the simulator car seat transfer tests. The 95% confidence intervals are shaded in grey. |
| --- |
| 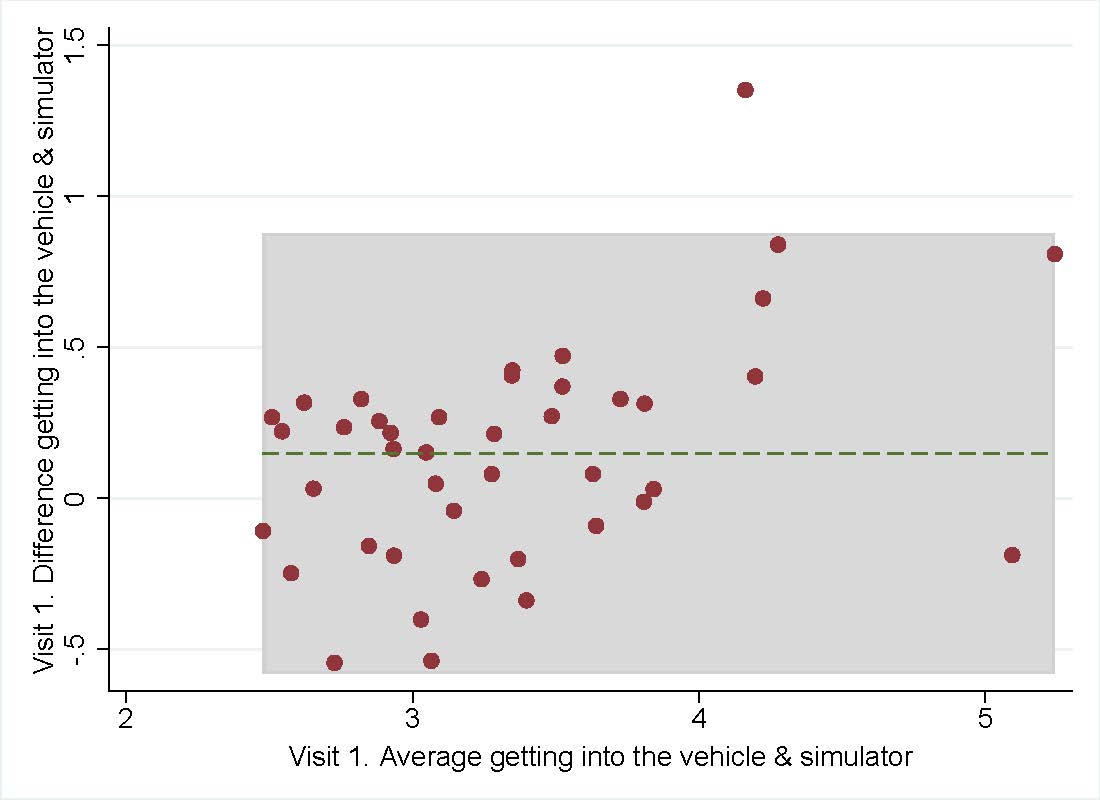 |

| **Supplement Figure 3.** Bland-Altman plots of the vehicle and the simulator getting out of the car seat transfer test times, reported in seconds, for the visit 1. The mean of the vehicle and the simulator getting into the car seat transfer test times was plotted against the difference of the vehicle and the simulator getting into the car seat transfer test times for each participant.  The centre line displays the mean difference for the participants between the vehicle and the simulator car seat transfer tests. The 95% confidence intervals are shaded in grey. |
| --- |
| 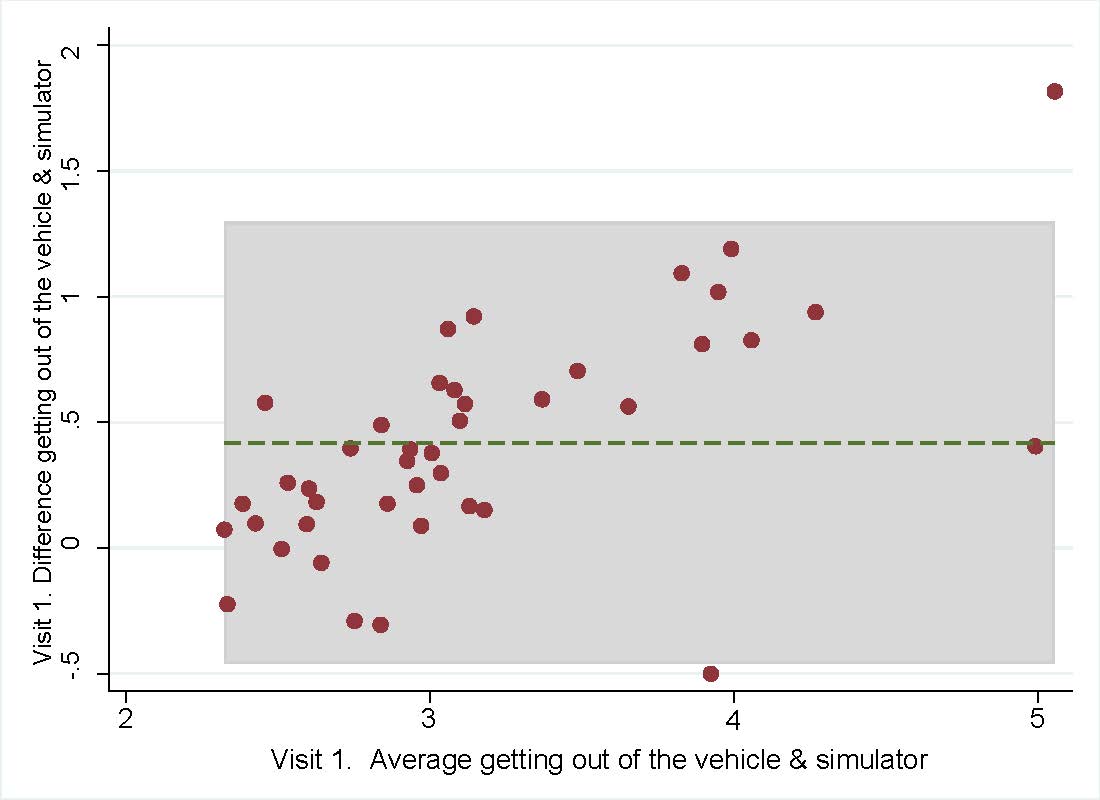 |

| **Supplement Figure 4** Bland-Altman plots of the vehicle and the simulator getting into the car seat transfer test times, reported in seconds, for the visit 2. The mean of the vehicle and the simulator getting into the car seat transfer test times was plotted against the difference of the vehicle and the simulator getting into the car seat transfer test times for each participant.  The centre line displays the mean difference for the participants between the vehicle and the simulator car seat transfer tests. The 95% confidence intervals are shaded in grey. |
| --- |
| 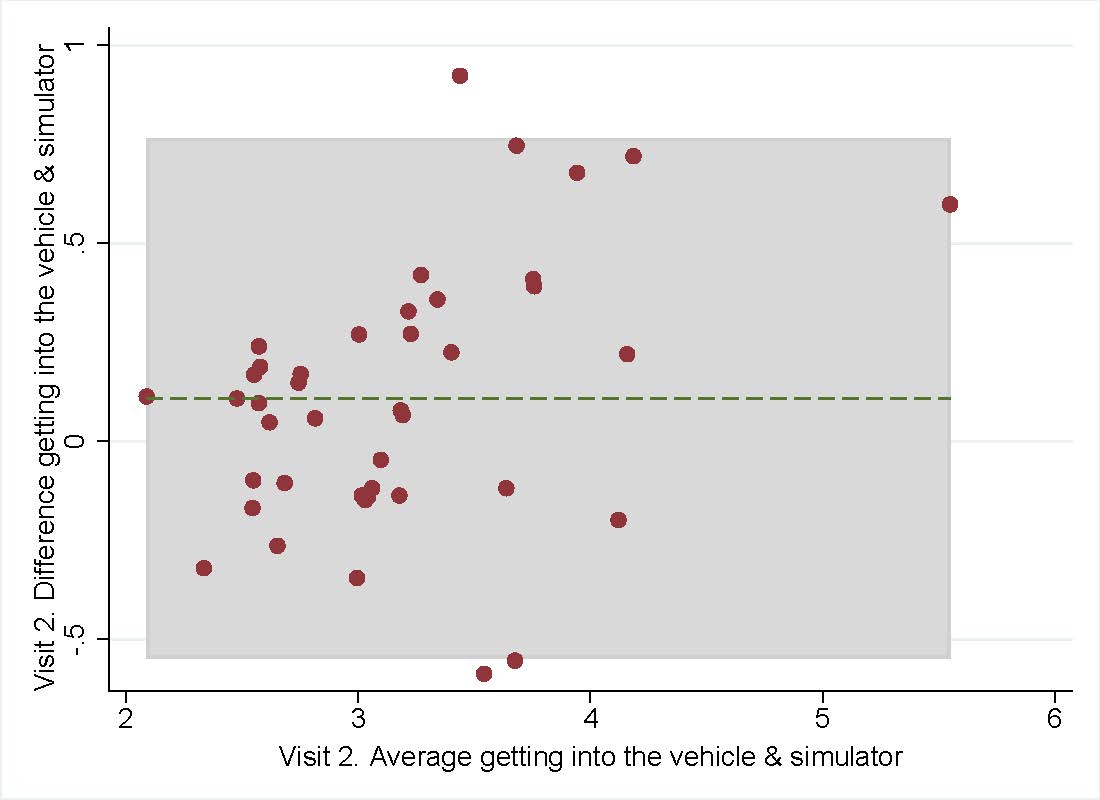 |

| **Supplement Figure 5.** Bland-Altman plots of the vehicle and the simulator getting out of the car seat transfer test times, reported in seconds, for the visit 2. The mean of the vehicle and the simulator getting into the car seat transfer test times was plotted against the difference of the vehicle and the simulator getting into the car seat transfer test times for each participant.  The centre line displays the mean difference for the participants between the vehicle and the simulator car seat transfer tests. The 95% confidence intervals are shaded in grey. |
| --- |
| 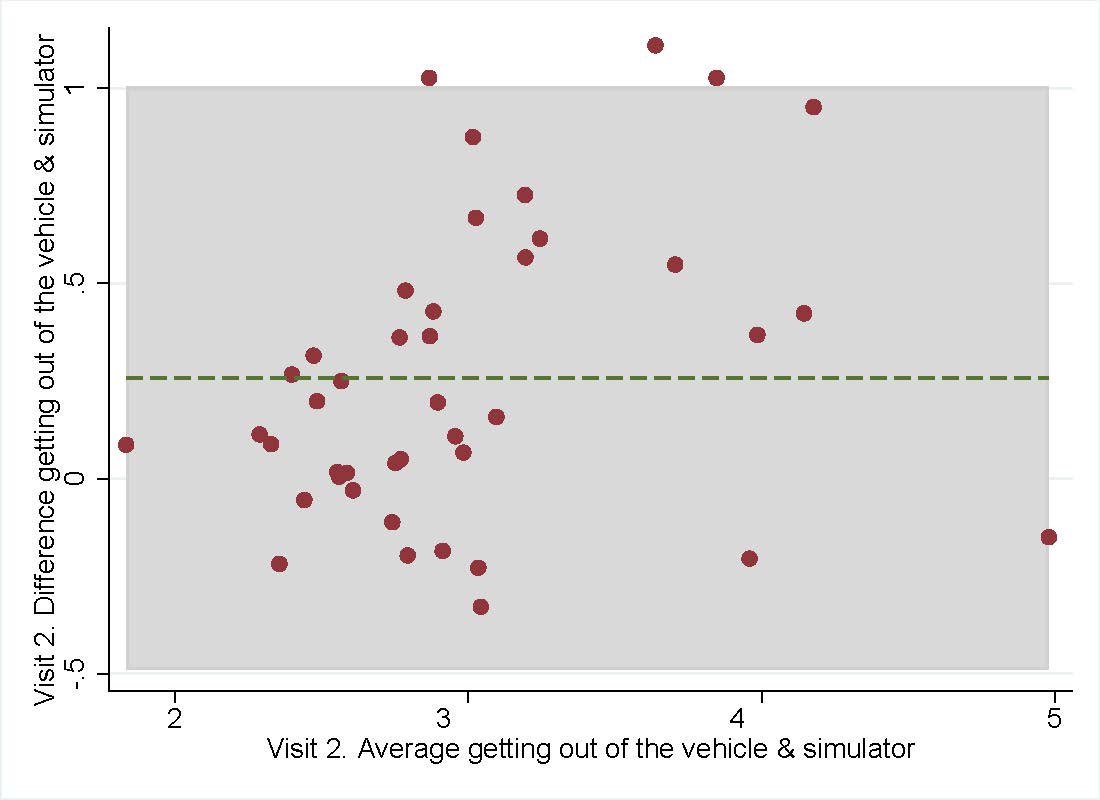 |
